# The weekend effect – How can it be mitigated? Introduction of a consultant-delivered emergency general surgical service

**DOI:** 10.1016/j.amsu.2020.08.013

**Published:** 2020-08-14

**Authors:** Khevan Somasundram, Jonathan J. Neville, Yashashwi Sinha, Tushar Agarwal, Durgesh Raje, Ashish Sinha, Hemant Sheth

**Affiliations:** a Ealing Hospital, London North West University Healthcare NHS Trust, London, UK; b Imperial College Healthcare NHS Trust, Paddington, London, UK; c University Hospitals Birmingham NHS Foundation Trust, Birmingham, UK

**Keywords:** Weekend effect, Emergency laparotomy, Emergency surgery

## Abstract

**Background:**

Poorer patient outcomes for emergency general surgery have been observed in patients admitted to hospital over the weekend. This paper reports the outcomes of a Consultant-delivered service model for weekend admissions and its impact for patients undergoing emergency laparotomy.

**Methods:**

Operative data was analysed from a prospectively collected database over 5-years. Primary outcome measures were 30-day all-cause mortality and Clavien-Dindo class ≥2 morbidity. Secondary outcomes included time from admission to diagnostic imaging and time to surgery, post-operative length of stay and requirement for Intensive Care Unit admission.

**Results:**

263 patients underwent an emergency laparotomy. Overall 30-day mortality was 4.6% and all-cause morbidity was 55.9%. The most common indications for laparotomy were mechanical small bowel obstruction (32.7%) and hollow viscus perforation (30.4%) of the 263 emergency laparotomies, 92 patients in the cohort were weekend admissions (Saturday or Sunday). There was no significant difference amongst patients admitted during the weekend in ASA grade, age, gender, or proportion of patients receiving a pre-operative computed tomography scan, when compared to those during the week. Compared to weekdays, weekend admission was not associated with a significant difference in mortality (5.3% and 3.3%, respectively p = 0.458), all-cause morbidity (p = 0.509), post-operative length of stay (p = 0.681), or Intensive Care Unit admission (p = 0.761).

**Conclusion:**

A Consultant Surgeon delivered emergency service can avoid the poor patient outcomes associated with weekend admissions and the ‘weekend effect’.

## Highlights

• Poorer patient outcomes have been observed in patients admitted during the weekend.
• A Consultant Surgeon delivered model of service delivery ensures peri- and intra-operative Consultant presence for all emergency surgery.
• Under this model, there was no weekend effect observed.

## 1 Introduction

The ‘weekend effect’ refers to the poorer clinical outcomes in patients admitted to hospital during the weekend (WA) compared to weekdays (WDA) [[1], [2], [3], [4], [5], [6], [7], [8], [9]]. Many possible reasons have been suggested. Reduced levels of clinical and auxiliary staffing and limited access to resources during the weekend may contribute as significant logistical and capacity-related limitations [10,11]. With respect to specialist services, reduced access to interventional radiology, therapeutic and diagnostic endoscopy and lack of early senior clinician engagement in the patient pathway may lead to poorer outcomes for WA patients. This phenomenon appears ubiquitous amongst specialties and is well documented in both medical and surgical emergency admissions. Patient-related features, including disease severity, possible delayed presentation, as well as differing thresholds for admission have also been shown to influence decision-making when planning to undertake emergency surgery during the weekend [1,12].

The majority of National Health Service (NHS) hospitals currently have little elective activity over the weekend. Emergency admissions and in-patients requiring emergency care constitute the majority of the weekend caseload. A number of studies have observed a weekend effect for emergency surgical admissions [[13], [14], [15], [16], [17]]. However, large studies demonstrate disagreements on the presence of a weekend effect. A large matched cohort study of patients compared weekday and weekend admission for non-cardiac surgery and identified an increase in 30-day mortality (OR 1.05; 95% CI 1.00–1.11) for patients admitted during the weekend. But this effect disappeared when adjusting for the urgency of admission, which was not associated with the day of admission [18]. On the other hand, a meta-analysis reporting the outcomes of over seven-million procedures identified that the risk of mortality had a stepwise increase with each day of the week, where weekend admission for emergency surgery was associated with the greatest increase in mortality risk (OR 1.27; 95% CI 1.08–1.49) [14]. Large studies deliver an aggregate overview of results with higher heterogeneity, which may be due to inter-center differences in data coding, or the study populations served. Conflicting results diminish equipoise in service delivery and distort public perception. The factors that mitigate the ‘weekend effect’ cannot be identified without targeting sources of discrepancies in service delivery. This justifies reporting single centre data, of which a paucity of reports exist. This can then be used to directly compare models of delivery and their outcomes.

Outcome reporting for emergency laparotomies demonstrate inconsistencies in care provision across the NHS [19]. National variations in socio-economic factors including provision of primary care, severity of co-morbidities and socio-economic deprivation may contribute to explaining widely ranging morbidity and mortality rates [20]. This instigated an initiative to corroborate and collectivize data in the National Emergency Laparotomy Audit (NELA) in 2013. NELA has served as a national database to aid the standardization of the multi-disciplinary peri-operative assessment of patients undergoing emergency laparotomy. But prior to 2015, the annualized national 30-day mortality rate was above 11%, and has since reduced to 9.5% in 2018 [19]. National statistics have continued to demonstrate poor outcomes, which led the authors to develop a model of care to improve this result.

In the 2018 NELA report, inconsistent Consultant presence was most apparent out-of-hours and during the weekend. More than 99% of all patients in NELA had been seen in-person or were discussed with a Consultant Surgeon. However, 90% and 66% of patients had intra-operative Consultant Surgeon and Anaesthetist presence in and out-of-hours, respectively. More than 30% of >23,000 cases were performed out of hours. Importantly, patients who underwent surgery out-of-hours had a higher proportion of patients with a predicted risk of death >10% (55%), compared to in-hours operations (35%). The observed 30-day mortality for these high-risk patients was 25%. The presence of both Consultant Surgeon and Anaesthetist for patients with a predicted mortality >5% during the weekend averaged between 55 and 85%. This means Consultant presence is lower when a greater proportion of high-risk patients present [19].

The involvement of experienced decision-making also has an impact on post-operative critical care admission, which evidently demonstrated a bearing on subsequent clinical outcomes in the NELA report. Around 63% and 87% of patients with a mortality risk >5% and >10%, respectively, were directly admitted to critical care post-operatively. However, 3.4% of all patients had unplanned critical care admissions due to deterioration in the ward after surgery, or unplanned return to the operating theatre. This statistic has remained static across previous national reports [19]. These patients had a 30-day mortality rate of 17.5% and double the mean length of post-operative in-hospital stay (16 vs 30 days). Unplanned critical care admissions between hospitals varied between 0% and 36% [19]. The high mortality rate of unplanned admissions reinforces the need for collaborative pre-operative decision-making amongst experienced members of the team.

An analysis of the NELA data from 2014 to 2017 showed no effect of weekend admission on post-operative outcomes [21]. However, this study excluded patients undergoing laparotomies for appendicitis and acute cholecystitis, which are the most common indications for emergency general surgery. Patients who underwent surgery more than one week after admission were also excluded, therefore creating a potential bias.

The impetus to introduce a Consultant-delivered service model at our unit derived from systemic inconsistencies in service delivery, represented by the ‘weekend effect’ and its compounding impact after emergency surgery. The authors advocate that patient outcomes hinge on experienced decision making, which is most accurately represented by detailed single-center data. This can aid comparisons with national data sets, and may serve to facilitate our model's wider adoption to aid its validation.

The aim of this study is to report the outcomes of emergency laparotomies between the weekday and weekend under a Consultant-delivered emergency General Surgical service at a single district general hospital. As a service evaluation, these results have been compared to NELA, which serves as a national benchmark in the United Kingdom.

## 2 Methods

KS, JJN and YS collected operative data from a prospectively maintained database between 1^st^ August 2014 and 31^st^ May 2019 at a 350-bed district general hospital. The collected data was validated by the surgeons who operated on patients in this cohort (HS, AS, TA and DR). Any uncertainties with the collected data were discussed. All emergency laparotomy cases for general surgery-related indications were included in the study. Patients undergoing laparotomy for Gynaecological or Urological indications were excluded after a post hoc analysis. Patient demographics and indication for surgery were documented, as well as the day of admission, day of operation, all ICU admissions and total length of stay (LOS). Patients admitted on a Saturday or Sunday were grouped as ‘weekend admissions’ (WA). This study has been designated as a service evaluation. Our centre contributes to the annual national data collection for NELA. We have extracted our data from the database to perform a comparative analysis to national statistics. This paper is registered in the Research Registry (Registration ID: researchregistry5652).

The primary outcome measures were 30-day all cause mortality and morbidity with complications determined by a Clavien-Dindo class ≥2 [22]. Secondary outcome measures included time from admission to diagnostic computer tomography (CT), time from admission to operation and diagnosis on CT scanning.

A subgroup analysis was performed for the 129 patients who underwent a laparotomy and were 65 years of age or older, forming 49% of study population. This age cutoff was chosen as patients over 65 undergoing emergency laparotomy have been shown to have an increased risk of mortality [23]. An additional subgroup analysis was performed for patients admitted to the ICU post-operatively, in which LOS and mortality rates were compared to patients who recovered on the Surgical ward.

Data were analysed using SPSS version 24 (IBM). Statistical significance level was determined at p ≤ 0.05. Categorical data was compared using the Pearson Chi-squared test. Parametric continuous datasets were compared using the T-test. Non-parametric datasets were analysed using the Mann-Whitney *U* test. Predictors of morbidity and mortality were analysed using binomial logistic regression and LOS was analysed using multiple linear regression. This work has been reported in line with the STROCCS criteria [24].

The catchment area of our unit covers North West London, constituting a large proportion of ethnic minority populations and higher deprivation. This area is shared with one other regional emergency general surgical service. The service at our hospital is staffed with 6 Consultant Surgeons, a 5-bed post-operative recovery unit with a one-to-one nursing ratio, and an 8-bed Intensive Care Unit (ICU) that has continuous Consultant Intensivist oversight. There are two 30-bedded dedicated surgical wards.

**Fig. 1:**
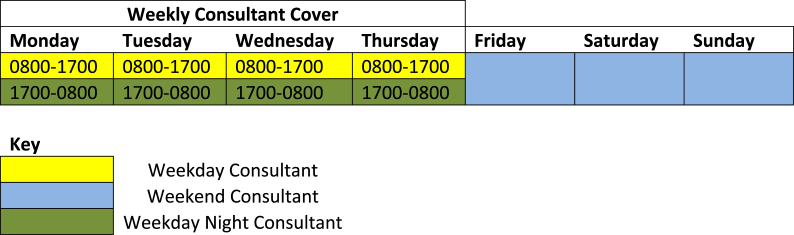
Outline of weekly on-call Consultant Surgeon cover.

The model of service delivery implemented since 2014 at our unit is summarized in Fig. 1. A Consultant Surgeon is on-take from Monday-Thursday, 8am-4pm. This weekday Consultant reviews all new patients admitted during this period on daily ward rounds. A second Consultant performs a daily evening ward round (7–9pm) for patients admitted after 5pm that day. Patients requiring a Consultant evening review are either handed-over from the day Consultant, or unwell patients are highlighted by the surgical resident prior to the evening ward round. The night Consultant provides non-resident out-of-hours cover till 8am the following day. During the weekend, the Consultant Surgeon is on-take for 72 h, from Friday-Monday morning. The weekend Consultant Surgeon performs twice-daily ward rounds. All newly admitted patients are reviewed by the on-call Consultant Surgeon within 12–14 h of admission.

The Consultant Surgeon on-call is free from any elective commitments. This ensures full engagement with the emergency caseload. Early Consultant oversight is present for the initial assessment and treatment, arrangement of diagnostic investigations and delivery of operative intervention with surgical residents if required. Decisions on resuscitation in the event of cardiac arrest are also clearly documented. The decision to operate is decided by the Consultant Surgeon with National Confidential Enquiry into Patient Outcome and Death (NCEPOD) [25] guidance and is contingent on the pre-operative anaesthetic review.

There is one dedicated operating theatre with accompanying staff for the emergency surgical list, which remains constant throughout the week. This list is shared with emergency Orthopaedic and Gynaecology services. Cases are prioritized in accordance to operative urgency graded by the NCEPOD scale. During weekdays, a dedicated Surgical resident (specialist registrar) is on-call to receive referrals from Monday-Thursday, 8am-8pm. The weekend resident provides cover from Friday-Monday, 8am-8pm. A night Surgical resident is present from 8pm to 8am from Monday-Friday and Friday-Monday morning. The anaesthetics team has an on-call resident that mirrors this schedule. At our district general hospital, residents that join our service as they rotate between Hospitals in the proximate geographical area are junior surgical trainees (equivalent of 1st-3rd year residents in the United States) that do not yet have the experience to undertake major emergency cases with indirect supervision or independently. Residents are expected to receive referrals and perform the initial assessment and resuscitation of patients. Of the patients requiring emergency surgery, the Consultant on-call is directly present in the peri- and intra-operative period. Residents function as first assistants in emergency operations and undertake the operation under direct Consultant guidance. Residents also perform rounds on newly admitted patients before the formal Consultant-led ward-round.

In the ICU, the nurse-to-patient ratio is maintained at one-to-one. On the surgical wards, one nurse is in charge of five patients. This is maintained throughout the week. An on-call nurse-led pain team is available in-hours. The pain team has a once-weekly ward round with a Consultant Anaesthetist for post-operative patients. The on-call resident anaesthetist takes out-of-hours pain-related enquiries. Physiotherapy teams are on-site daily throughout the week and engage with patients within the first day after surgery. An out-of-hours chest physiotherapy service is available. Specialist dietetic services are available during weekdays and are also core members of the multi-disciplinary team.

## 3 Results

**Fig. 2:**
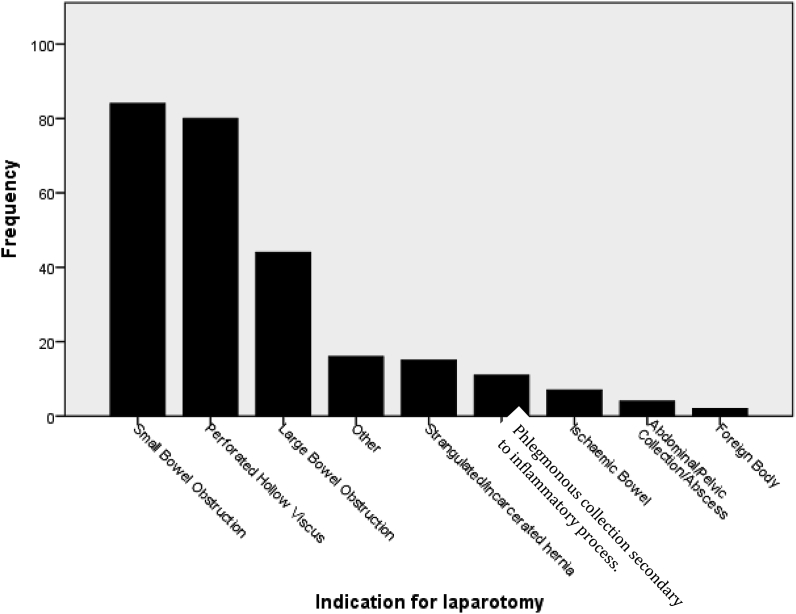
Relative frequencies of indications for emergency laparotomy for all 263 procedures. The 80 emergency laparotomies performed for a perforated hollow viscus were due to: perforations of the large bowel (27), duodenum (26), appendix (10), stomach (nine), small bowel (four), Meckel's diverticulum (one), and rectum (one). The culprit perforated viscus was unspecified in two cases. Other indications included: haemoperitoneum (eight), exploratory laparotomy (two), splenic rupture (two), common bile duct leak (one), fulminant ulcerative colitis (one), colovesical fistula (one) and retained pillcam (one).

Some 263 patients underwent an emergency laparotomy, comprising of 171 (65%) weekday and 92 (35%) weekend admissions. 147 (55%) patients were male. Overall 30-day mortality was 4.6% and 30-day all-cause Clavien-Dindo class ≥2 morbidity was 55.9%. A pre-operative abdominal CT scan was performed in 211 (80%) patients. Between WA versus WDA, there was no significant difference in time from admission to imaging (20.6 ± 48.0 h vs 15.6 ± 36.0 h p = 0.432) or procedure (2.44 ± 4.6 days vs 1.5 ± 2.5 days p = 0.063). The most common indications for emergency surgery were small bowel obstruction (33%), perforated abdominal viscus (30%) and large bowel obstruction (13%) (Fig. 2).

There was no significant difference between WDA and WA groups when considering patient demographics - median patient age (Inter-quartile range) (66 (46–78) years and 58 (42.8–78) years, respectively p = 0.135); gender (54.3% female, 58.7% male, p = 0.502) or median ASA grade (2 and 2, respectively p = 0.226). The indication for surgery based on clinical examination and CT scan results did not also significantly differ between the two groups. Thirty-day mortality was also not significantly different between the two groups. Neither was all-cause 30-day morbidity. Rates of re-operation, re-admission, ICU admission and median LOS did not demonstrate a significant difference between the two groups (Table 1).

**Table 1:** Comparison of patient characteristics and incidence of post-operative complications for patients admitted for emergency laparotomy during the weekday compared to weekend admissions.

		Weekday Admission (n = 171)	Weekend Admission (n = 92)	P-value
Age	Mean (SD)	61.9 (±20.0)	58.1 (±20.8)	0.135*
	Median (Q1 – Q3)	66 (46–78)	58 (42.8–78)	0.175**
Gender	Male (%)	93 (54.3)	54 (58.7)	0.502***
	Female (%)	78 (45.6)	38 (41.3)	
ASA grade	Median (Q1 – Q3)	2 (1–2)	2 (1–2)	0.226**
Indication	Small Bowel Obstruction	53	31	0.620***
	Perforated Hollow Viscus	48	32	
	Large Bowel Obstruction	33	11	
	Other	11	5	
	Strangulated/Incarcerated Hernia	9	6	
	Phlegmon	9	2	
	Ischaemic Bowel	4	3	
	Abdominal/Pelvic Collection	2	2	
	Foreign Body	2	0	
Time to imaging (hours)	Mean	20.6 (±48.0)	15.6 (±36)	0.432*
Time from imaging to operation (days)	Mean	1.3 (±2.3)	1.1 (±2.1)	0.436*
Time from admission to operation (days)	Mean	2.44 (±4.6)	1.5 (±2.5)	0.063*
Length of stay (days)	Mean	17.3 (±13.0)	16.6 (±12.1)	0.681*
	Median (Q1 – Q3)	12.5 (8–22)	12 (9–20.5)	0.960**
Mortality (30-day)	All-cause	9	3	0.458***
	Sepsis (unknown source)	4	0	
	Pneumonia (aspiration/HAP)	2	2	
	Cardiac event	1	1	
	Metastatic cancer	1	0	
	Ischaemic bowel	1	0	
Morbidity (30-day)	All-cause	94	53	0.509***
	HAP	28	19	0.388***
	Pulmonary embolism	2	0	0.298***
	Cardiac event	9	7	0.448***
	Cerebrovascular accident	1	0	0.462***
	Culture-positive bacteraemia	5	5	0.310***
	Deep vein thrombosis	1	1	0.655***
	Post-operative Intra-abdominal collection	16	14	0.196***
	Wound Infection	13	12	0.151***
	Re-operation	7	4	0.744***
	Re-admission	19	18	0.083***
	Intensive care unit admission	40	20	0.761***

*T-Test (two tailed). **Mann-Whitney *U* Test. ***Pearson Chi-squared Test. Abbreviations: standard deviation (SD), interquartile range (IQR), hospital-acquired pneumonia (HAP).

Patients undergoing laparotomy for appendicectomy and cholecystectomy were excluded (n = 22) to compare outcomes with NELA data [19]. In this sub-group analysis, the overall all-cause 30-day morbidity and mortality rates were 56.4% and 5.0%, respectively. The median ASA grade for the cohort excluding laparotomies for appendicectomy and cholecsytectomy was 2 (1–2).

Relating to the day of surgery, 180 operations were performed on a weekday and 83 during the weekend. Of the weekend procedures, 34 (41%) operations were performed on patients admitted during the weekend of presentation. The remaining weekend operations were performed on patients admitted earlier in the week. Thirty-two of 180 weekday operations were performed on the Monday after admission during the preceding weekend. Between weekday and weekend operations, there was no significant difference in age (p = 0.089), gender (p = 0.667), median ASA grade (p = 0.201), or indication for surgery (p = 0.096). Emergency laparotomies performed during the weekend did not result in a significantly different 30-day mortality (4.8% vs 4.4% p = 0.892) or morbidity rate (57.8% vs 55.6% p = 0.656). There was also no difference in re-operation (p = 0.500) or re-admission rates (p = 0.449). Ten of 283 patients developed post-operative blood culture-proven bacteraemia. Six of these patients had their operation on a weekend and four underwent surgery during a weekday, a result that demonstrated a statistically significant difference (p = 0.048). Of the six patients who developed a bacteraemia after a weekend operation, three underwent surgery during the same weekend of admission. The remaining three were admitted earlier in the week.

For the subgroup analysis of patients aged 65 years or older, 38 (30%) of 129 patients were WA. There was no significant difference during the weekend in mean age (p = 0.553), gender (p = 0.708), median ASA grade (p = 0.567) or indication for surgery (p = 0.847) in this subgroup. The 30-day mortality rates for these WDA and WA subgroups were 6.6% and 8%, respectively (p = 0.791). No significant differences in all-cause morbidity (p = 0.165) or LOS (p = 0.557) were detected.

The mortality rates for WDA and WA patients admitted to the ICU post-operatively were 5.0% and 7.5%, respectively (p = 0.714). Irrespective of WDA or WA, there was no difference in 30-day mortality between patients admitted to the ICU post-operatively, compared to patients who recovered on the ward (6.7% vs 4.0%, respectively p = 0.591). The median post-operative LOS for all patients admitted to the ICU was significantly longer, compared to patients who did not require ICU admission (17.5 days (11–29) vs. 12 days (8–18), respectively p= <0.001).

Odds ratios (OR) were calculated for weekday admissions using a binomial logistic regression model compared with the whole cohort (Table 2). Admission on a weekday was associated with an OR of 0.69 (p = 0.602) for 30-day mortality and 1.44 (p = 0.194) for 30-day all-cause morbidity. Increased age was associated with an increased risk of morbidity, OR 1.03 (1.01–1.04, p = 0.001), while a higher ASA grade portended a higher risk of mortality OR 4.26 (1.52–11.90, p = 0.006).

**Table 2:** Binomial logistic regression for 30-day mortality and morbidity.

	30-day Mortality*		30-day all-cause Morbidity**	
	OR (95% CI)	p-value	OR (95% CI)	p-value
Admission on Weekday	0.69 (0.17–2.84)	0.602	1.44 (0.83–2.48)	0.194
Age	1.03 (0.99–1.08)	0.145	1.03 (1.01–1.04)	*0.001*
Male Gender	0.23 (0.05–1.17)	0.077	0.90 (0.52–1.55)	0.700
Mean ASA Grade	4.26 (1.52–11.90)	*0.006*	1.37 (0.89–2.10)	0.152
Time from admission to procedure	0.88 (0.70–1.11)	0.268	1.06 (0.98–1.15)	0.170

*Model was significant at p < 0.001. Nagelkerke R^2^ = 0.257. **Model was significant at p = 0.023. Nagelkerke R^2^ = 0.126.

## 4 Discussion

This study aimed to describe a Consultant-delivered emergency service model. Specifically, this model was assessed to discern if disparities in outcomes exist between patients admitted for emergency laparotomy during the weekend and weekdays over a 5-year period.

Admission during the weekend was not observed to result in an increased 30-day morbidity or mortality rate. This cohort did not demonstrate a significant difference in patient characteristics presenting throughout the week. Operating on a weekend was not associated with a higher all-cause morbidity or mortality rate, but did reveal higher incidence of post-operative bacteraemia. However, these were small numbers that resulted in a marginal statistically significant difference.

This study was undertaken on the background of poor national outcomes after emergency laparotomy and large studies that demonstrated conflicting results on the presence of the ‘weekend effect’. Using aggregate data, this has created uncertainty in the efforts to strategize healthcare delivery to uniformly rectify this observation. By identifying root causes of discrepancies in service delivery to explain the heterogeneity of data, this will facilitate the generation of policy solutions. With the premise that direct intra- and peri-operative Consultant oversight of the team is central to the model described in this study, we advocate that good outcomes can be achieved. Moving forward, a way to validate this is to directly compare models of delivery and clinical outcomes in similarly sized centers over wider geographical areas with differing patient demographics. The adoption of this model will allow centers to compare their outcomes before and after changes in their services to demonstrate if the results in this study can be replicated.

The absolute 30-day mortality in our cohort was lower than that reported in the 2017 NELA data (5.0% versus 9.5%) [19]. While 95% and 86% of NELA cases had Consultant Surgeon and Anaesthetist involvement in the decision to operate, 78% of all cases had both Consultants present intra-operatively. Amongst patients with a predicted mortality >10%, 86% had intra-operative Consultant Surgeon and Anaesthetist presence. Importantly, the observed 30-day mortality amongst patients with the highest operative risk was 20% [19]. In our study, Consultant input and intra-operative presence was present for 100% of patients. This model is also supported by our Radiology colleagues, who ensured minimal delay in access to diagnostic imaging and its subsequent prompt reporting by a Consultant Radiologist. Pre-operative engagement from senior Anaesthetists and Critical Care teams aid in case selection and decisions about post-operative destination, therefore limiting unplanned critical care admissions. All ICU admissions were grouped in this study and our results expectedly demonstrate these patients have a longer in-hospital stay post-operatively. This Consultant-delivered rather than Consultant-led model may explain the lower absolute mortality rate and absence of the ‘weekend effect’ in this study.

The incipient trend of Consultants taking on the role of front-line providers has been proposed to aid reforms in service provision, likely due to the poor outcomes for emergency surgical care observed nationally [19,26]. Acute general surgical admissions have increased by 30% over the past decade [26]. This necessitates effective decision-making to confront the emergency caseload while balancing this with elective workload [25]. The challenge of determining risk is reflected in this study, in which Consultants have direct involvement in the pre-operative risk assessment and case selection. 18% of our cohort had an ASA grade ≥3, compared to 54% of patients from the NELA report [19]. There was appropriate risk stratification early on in the patient pathway and senior involvement in the decision to operate and when the operation should take place. This ensured that only patients who were expected to have an appropriate post-operative outcome were selected to undergo major surgery [27].

### 4.1 Limitations

An important limitation of this study is the relatively small, longitudinal patient cohort from a single unit. This report's external validity and model of service delivery is therefore arguably limited to other centers of a similar size, resource capacity and annual emergency caseload. There were no data on the number of patients deemed unfit, or those who declined emergent surgical intervention. Although age, ASA grade and indication for surgery were not different between weekend and weekday admissions, selection bias for patients who undergo surgery may confound this study's observations by only including patients that had an operation. The authors advocate that this consultant-delivered model and its encompassing interventions provide consistent, high-quality care. However, a comparison to patient outcomes prior to changes in the service delivery model has not been considered to prove causation of these results.

## 5 Conclusion

Weekend admission for emergency laparotomy was not associated with increased 30-day morbidity or mortality. Under a Consultant-delivered emergency service model, no weekend effect was observed and absolute mortality rates were lower than that of the NELA data. This work can be taken forward by identifying patient and logistical variables that better predict adverse outcomes and comparing this to predictions made by standardized risk models. The validation of this study will require a further assessment of patient outcomes with greater numbers, as well as a comparison to other models of service delivery in centers of a similar size and emergency service caseload.

## Funding

This research did not receive any specific grant from funding agencies in the public, commercial, or not-for-profit sectors. The datasets analysed for this study are available from the corresponding author on reasonable request.

## Ethical approval

No ethical approval was received for this research. This work is designated as a service evaluation. All data was extracted from a national database.

## Research registration unique identifying number (UIN)

1. Name of the registry: **Research Registry**
2. Unique Identifying number or registration ID: **researchregistry5652**
3. Hyperlink to your specific registration (must be publicly accessible and will be checked): https://www.researchregistry.com/browse-the-registry#home/registrationdetails/5ed007e70e00fc0015c6f74d/

## Guarantor

Khevan Somasundram.

Hemant Sheth.

## Provenance and peer review

Not commissioned, externally peer reviewed.

## CRediT authorship contribution statement

**Khevan Somasundram:** Data collection, Investigation, Methodology, Data interpretation, Formal analysis, Conceptualization, Writing - original draft. **Jonathan J. Neville:** Data collection, Investigation, Methodology, Formal analysis, Writing - original draft. **Yashashwi Sinha:** Data collection, Formal analysis, Writing - review & editing. **Tushar Agarwal:** Validation, Supervision, Writing - review & editing. **Durgesh Raje:** Validation, Supervision, Writing - review & editing. **Ashish Sinha:** Validation, Supervision, Writing - review & editing. **Hemant Sheth:** Conceptualization, Validation, Supervision, Writing - review & editing, Project administration.

## Declaration of competing interest

None.

## References

[1] Freemantle N., Richardson M., Wood J., Ray D., Khosla S., Shahian D. (2012 Feb). Weekend hospitalization and additional risk of death: an analysis of inpatient data. J. Roy. Soc. Med..

[2] Aylin P., Alexandrescu R., Jen M.H., Mayer E.K., Bottle A. (2013 May). Day of week of procedure and 30 day mortality for elective surgery: retrospective analysis of hospital episode statistics. BMJ.

[3] Aylin P., Yunus A., Bottle A., Majeed A., Bell D. (2010 Jun). Weekend mortality for emergency admissions. A large, multicentre study. Qual. Saf. Health Care.

[4] Ricciardi R., Roberts P.L., Read T.E., Baxter N.N., Marcello P.W., Schoetz D.J. (2011 May). Mortality rate after nonelective hospital admission. Arch. Surg..

[5] Barba R., Losa J.E., Velasco M., Guijarro C., Garcia de Casasola G., Zapatero A. (2006 Aug). Mortality among adult patients admitted to the hospital on weekends. Eur. J. Intern. Med..

[6] Handel A.E., Patel S.V., Skingsley A., Bramley K., Sobieski R., Ramagopalan S.V. (2012). Weekend admissions as an independent predictor of mortality: an analysis of Scottish hospital admissions. BMJ Open.

[7] Ruiz M., Bottle A., Aylin P.P. (2015 Aug). The Global Comparators project: international comparison of 30-day in-hospital mortality by day of the week. BMJ Qual. Saf..

[8] Mohammed M.A., Sidhu K.S., Rudge G., Stevens A.J. (2012 Apr). Weekend admission to hospital has a higher risk of death in the elective setting than in the emergency setting: a retrospective database study of national health service hospitals in England. BMC Health Serv. Res..

[9] Lee K.G., Indralingam V. (2012 Oct). A study of weekend and off-hour effect on mortality in a public hospital in Malaysia. Med. J. Malaysia.

[10] Scott J.W., Tsai T.C., Neiman P.U., Jurkovich G.J., Utter G.H., Haider A.H. (2018 Mar). Lower emergency general surgery (EGS) mortality among hospitals with higher-quality trauma care. J. Trauma Acute Care Surg..

[11] Ozdemir B.A., Sinha S., Karthikesalingam A., Poloniecki J.D., Pearse R.M., Grocott M.P.W. (2016 Jan). Mortality of emergency general surgical patients and associations with hospital structures and processes. Br. J. Anaesth..

[12] Mikulich O., Callaly E., Bennett K., O'Riordan D., Silke B. (2011). The increased mortality associated with a weekend emergency admission is due to increased illness severity and altered case-mix. Acute Med..

[13] Hoehn R.S., Go D.E., Dhar V.K., Kim Y., Hanseman D.J., Wima K. (2018 Feb). Understanding the ‘weekend effect’ for emergency general surgery. J. Gastrointest. Surg..

[14] Smith S.A., Yamamoto J.M., Roberts D.J., Tang K.L., Ronksley P.E., Dixon E. (2018 Feb). Weekend Surgical Care and Postoperative Mortality: A Systematic Review and Meta-Analysis of Cohort Studies. Med Care. https://www.ncbi.nlm.nih.gov/pubmed/29251716.

[15] Thomas C.J., Smith R.P., Uzoigwe C.E., Braybrooke J.R. (2014 Mar). The weekend effect: short-term mortality following admission with a hip fracture. Bone Joint J..

[16] Nandyala S.V., Marquez-Lara A., Fineberg S.J., Schmitt D.R., Singh K. (2013 Dec). Comparison of perioperative outcomes and cost of spinal fusion for cervical trauma: weekday versus weekend admissions. Spine.

[17] Ananthakrishnan A.N., McGinley E.L. (2013 May). Weekend hospitalisations and post-operative complications following urgent surgery for ulcerative colitis and Crohn's disease. Aliment Pharmacol. Ther..

[18] O'Leary J.D., Wunsch H., Leo A.-M., Levin D., Siddiqui A., Crawford M.W. (2019 Jan). Hospital admission on weekends for patients who have surgery and 30-day mortality in Ontario, Canada: a matched cohort study. PLoS Med..

[19] NELA Project Team (2018). Fourth Patient Report of the National Emergency Laparotomy Audit.

[20] Poulton T.E., Moonesinghe R., Raine R., Martin P., Anderson I.D., Bassett M.G., Cromwell D.A., Davies E., Eugene N., Grocott M.P., Johnston C. (2020 Jan 1). Socioeconomic deprivation and mortality after emergency laparotomy: an observational epidemiological study. Br. J. Anaesth..

[21] Nageswaran H., Rajalingam V., Sharma A., Joseph A.O., Davies M., Jones H. (2019 May). Mortality for emergency laparotomy is not affected by the weekend effect: a multicentre study. Ann. Roy. Coll. Surg. Engl..

[22] AssesSurgery Gmbh (2020). The Clavien-Dindo Classification. https://www.assessurgery.com/clavien-dindo-classification/.

[23] Cook T.M., Day C.J.E. (1998). Hospital mortality after urgent and emergency laparotomy in patients aged 65 yr and over. Risk and prediction of risk using multiple logistic regression analysis. Br. J. Anaesth..

[24] Agha R., Abdall-Razak A., Crossley E., Dowlut N., Iosifidis C., Mathew G., for the STROCSS Group (2019). The STROCSS 2019 guideline: strengthening the reporting of cohort studies in surgery. Int. J. Surg..

[25] (2020). National Confidential Enquiry into Patient Outcome and Death. NCEPOD. United Kingdom: NCEPOD.

[26] Rance C., Richards S.K., Jones A.E. (2016 May). Front door surgeons: the rise of consultant-delivered acute surgical care. Br. J. Gen. Pract..

[27] Hackett N.J., De Oliveira G.S., Jain U.K., Kim J.Y. (2015 Jun 1). ASA class is a reliable independent predictor of medical complications and mortality following surgery. Int. J. Surg..

